# Occupational Therapy Intervention for Intensive Care Unit-Acquired Weakness: A Case Report

**DOI:** 10.7759/cureus.87014

**Published:** 2025-06-30

**Authors:** Yusuke Aoyama, Akihito Yoshida

**Affiliations:** 1 Department of Rehabilitation, Graduate School of Medicine, Nagoya University, Nagoya, JPN; 2 Department of Integrated Health Sciences, Graduate School of Medicine, Nagoya University, Nagoya City, JPN

**Keywords:** activities of daily living, case report, functional independence measure, intensive care unit-acquired weakness, occupational therapy

## Abstract

Intensive care unit-acquired weakness (ICU-AW) poses a challenge to physicians and paramedical staff. ICU-AW causes long-term limitations in patients’ daily lives; therefore, we aimed to devise an effective treatment strategy. We present a novel occupational therapy strategy for patients with ICU-AW.

We present the case of a 52-year-old male with ICU-AW developed by cardiogenic shock. The patient was bedridden all day at the start of occupational therapy. To improve upper extremity activity time, enhance muscle strength, and promote activities of daily living (ADL), we used a portable spring balancer (PSB) to support arm movements, modified the nurse call to be thicker, adjusted the bed’s head-up angle, and collaborated with nursing staff to facilitate daily practice. Training was structured around repetitive, goal-directed upper extremity exercises both on a bed or on a wheelchair. Following the intervention, the patient demonstrated marked improvements in upper extremity strength and ADL, and regained independence in feeding and grooming.

This case report suggests that PSB-assisted, goal-directed OT combined with environmental modifications may be a safe and effective rehabilitation strategy even for patients with severe ICU-AW.

## Introduction

Intensive care unit-acquired weakness (ICU-AW), characterized by whole-body muscle weakness, is a complication of critical illness [1]. Risk factors for ICU-AW are multiple organ failure, hyperglycemia, corticosteroid use, neuromuscular blocker use, and muscle inactivity [2]. The prevalence of ICU-AW has been reported to range from 25% to 75%, with a median of 43% [3]. The incidence of ICU-AW increases significantly in patients receiving mechanical ventilation. ICU-AW occurs in 25-65% of patients ventilated for five to seven days [4]. ICU-AW is a known poor prognostic factor, associated with a prolonged hospital stay [4,5], increased risk of mortality [6], decline in activities of daily living (ADL) [7], and increased healthcare costs [4]. A propensity score-matched analysis showed that the one-year and five-year mortality risks following ICU discharge depend on the persistence and severity of muscle weakness. Patients with severe ICU-AW are at a particularly high risk of death [8]. Although the prevalence of ICU-AW gradually decreases after discharge (36% at discharge, 14% at one year, and 9% at two years), muscle strength improves over time. However, the six-minute walk test and SF-36 physical function scores remain impaired even after two years [3]. ICU-AW leads to a long-term decline in activities of daily living (ADL) after discharge [9], necessitating an effective treatment.

While electrical stimulation therapy is recommended for the prevention of ICU-AW, limited evidence exists for the therapeutic strategies after the onset of ICU-AW [10]. A rehabilitation guideline recommends early rehabilitation of ICU-AW [11]. The role of occupational therapy (OT) is to assess physical and mental functions, reduce/recover the amount of ADL assistance, and support ADL after discharge. Therefore, OT contributes to the improvement of ICU-AW through ADL-based strategies. In recent years, it has been reported that OT intervention strategies targeting ADL and physical and cognitive functions in the ICU have improved ADL outcomes [12,13]. However, scientific evidence was limited by the small number of studies [12]. Exploring effective intervention strategies and accumulating studies with a high level of evidence is needed.

In this case report, we used a portable spring balancer (PSB) (Hny International LLC, Tokyo, Japan) to extend the activity time of the upper extremities in a patient with ICU-AW. The patient showed improvements in both upper extremity muscle strength and ADL after the OT intervention. This report also aims to discuss intervention strategies.

## Case presentation

A 52-year-old male with dilated cardiomyopathy presenting with fatigue, loss of appetite, and orthopnea was admitted to the emergency department of another hospital. His body weight was 70.9 kg and height was 163.7 cm. He was on medication for heart failure, including carvedilol (Acharist®), enalapril (Lanirapid®), losartan (Cozaar®), furosemide (Lasix®), spironolactone (Aldactone®), tolvaptan (Samsca®), and pimobendan (Acardi®). Trichlormethiazide (Diart®) and potassium chloride (Slow-K®) were also prescribed to maintain volume and electrolyte balance. He had a history of heart failure, diagnosed eight years prior, at which time an implantable cardioverter-defibrillator was inserted. Four years ago, when his symptoms worsened to New York Heart Association (NYHA) functional class II-III, he received an additional cardiac resynchronization therapy defibrillator (CRT-D) implant. The patient had been managing his heart failure independently. He had no episodes of medication nonadherence and demonstrated good clinical familiarity and engagement in his care. The following day, the patient developed cardiogenic shock and was transferred to our hospital’s ICU under intubation, percutaneous cardiopulmonary support (PCPS), and intra-aortic balloon pumping.

**Figure 1:**
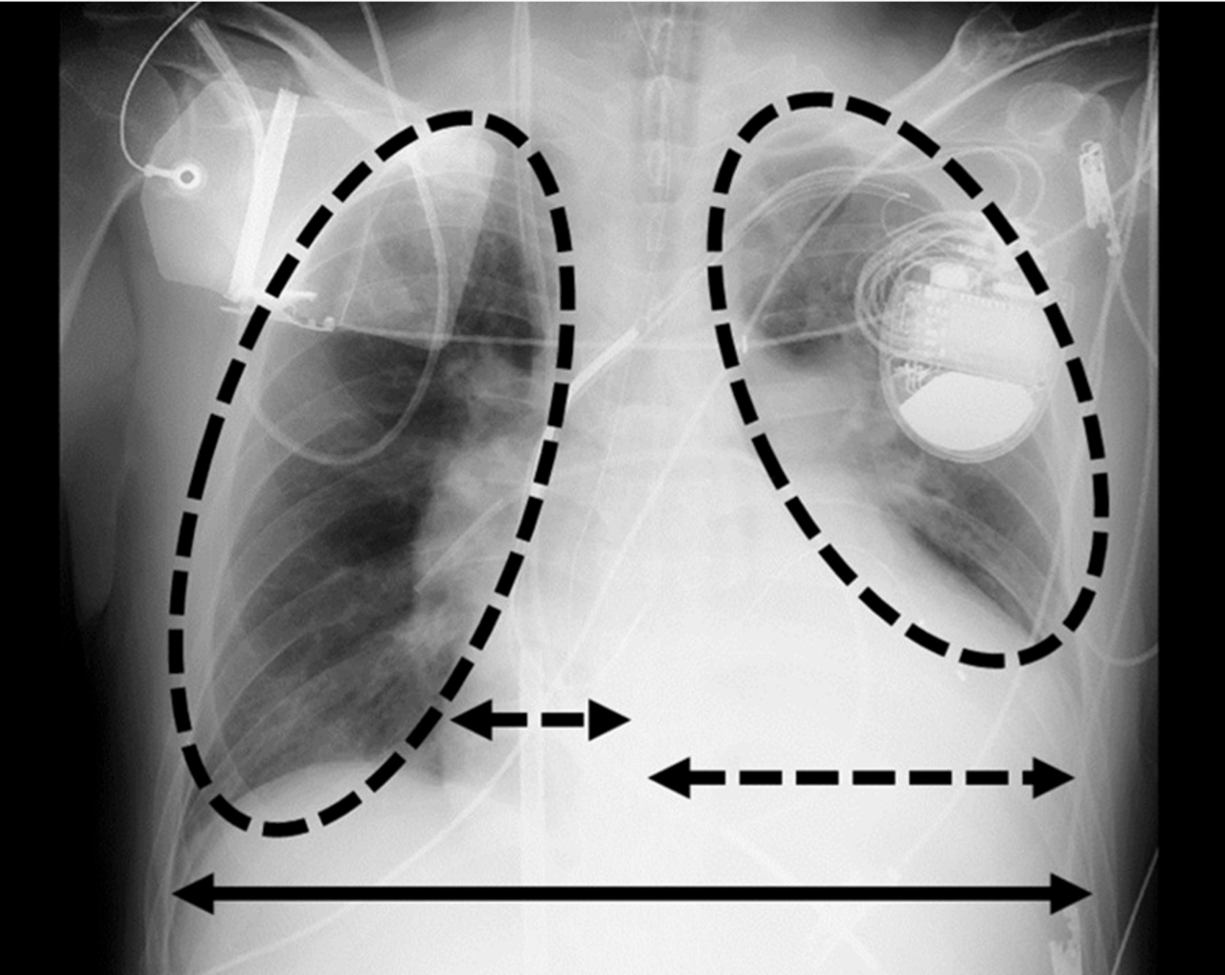
Frontal view of the chest X-ray on admission to our hospital (anteroposterior supine view). The right and left lung fields (circular dashed lines) showed reduced permeability. The cardiothoracic ratio calculated from the maximum transverse diameter of the lung fields (solid arrow) and the maximum transverse diameter of the heart (dashed arrow) in the figure was 66.6%.

The Acute Physiologic Assessment and Chronic Health Evaluation II (APACHE-2) score was 28 out of 71. The patient had impaired consciousness and peripheral cold sensation. Chest radiographs revealed left and right lung congestion and cardiomegaly (cardiothoracic ratio, 66.6%) (Figure 1). Echocardiography revealed morphological and functional declines: left ventricular ejection fraction 13.0%, left ventricular end-diastolic diameter/left ventricular end-systolic diameter 88/83 mm, interventricular septum/left ventricular posterior wall thickness 8.6/8.6 mm, and diffuse severe left ventricular systolic dysfunction. Blood gas analysis revealed the following: hydrogen ion concentration (pH) 7.408 and partial pressure of arterial oxygen (PaO_2_) 129.6 mmHg. Blood biochemistry results were as follows: white blood cell count 10.9x10^3^/µL, C-reactive protein (CRP) 18.4 mg/dL, hemoglobin 11.2 g/dL, prothrombin time-international normalized ratio 1.07, and albumin: 3.1 g/dL.

Poor aortic valve release was improved by the milrinone (Milrinone Injection Bag TAKATA®) 0.5γ and volume control. The patient presented with a fever and a severe inflammatory reaction at the time of transfer. The fever was possibly drug-induced and resolved when milrinone and amiodarone hydrochloride (amiodarone hydrochloride injection 150 mg TE®) were changed to olprinone hydrochloride hydrate (Coretec Injection®) and nifekalant hydrochloride (Shinbit Injection 50mg®), respectively. PCPS was weaned off on day 5. Extubating was conducted on day 9; however, the patient developed an unstable respiratory condition and a ventricular tachycardia (VT) storm on day 11. The patient underwent external ventricular assist device attachment on day 17, as well as physiotherapy (PT), OT, and speech therapy (ST) on days 19, 68, and 70, respectively. Due to hemodynamic instability and the use of sedatives, active rehabilitation was difficult; therefore, although the initiation of OT and ST was attempted multiple times, their initiation was delayed compared to PT.

**Figure 2:**
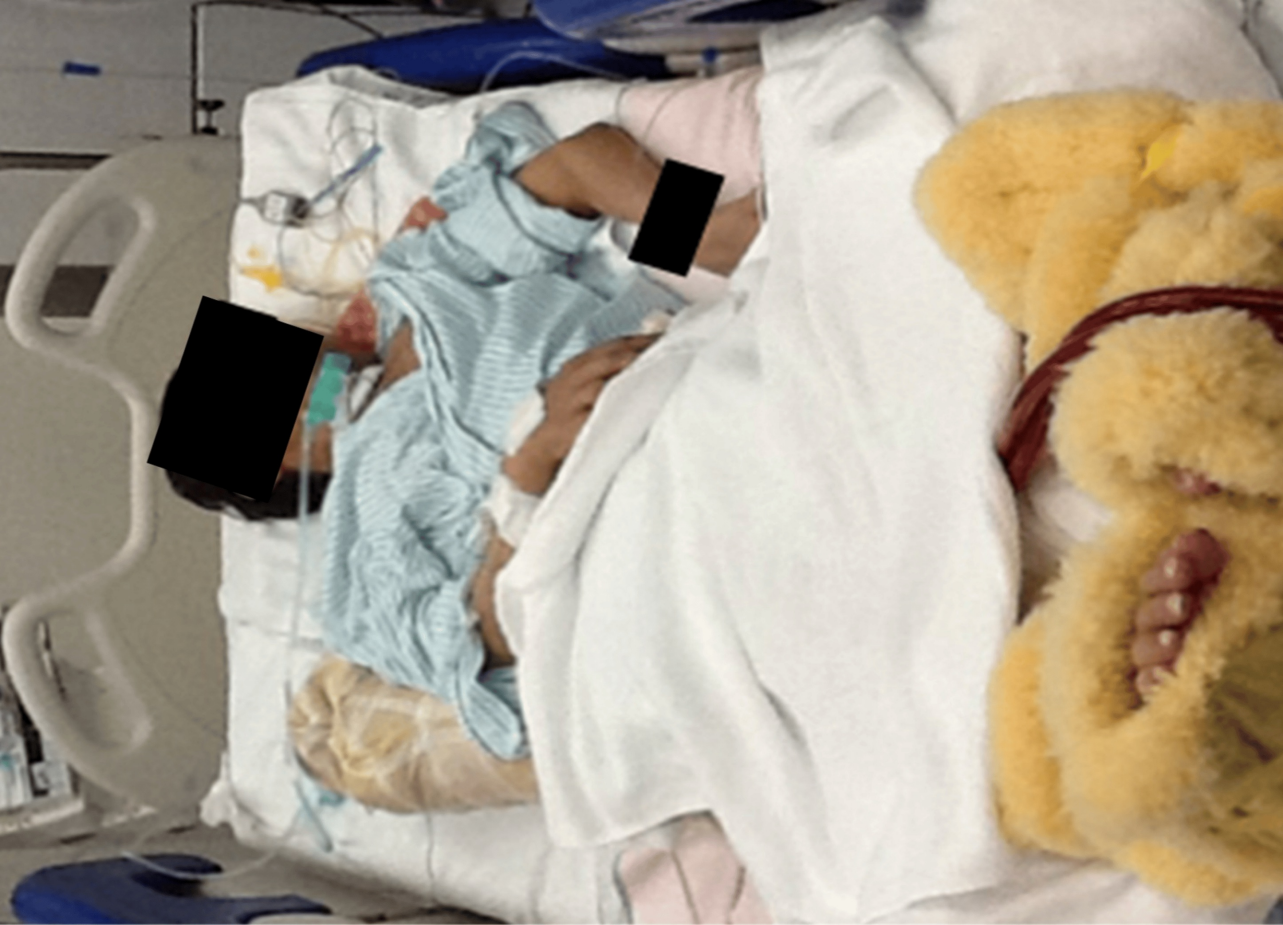
Bedridden patient at the start of the occupational therapy. Muscle weakness in the whole body was significant: Medical Research Council Score 6/30, grip and pinch strengths (right/left) 0/0 kgf, Functional Independent Measure self-care item score 6/42, active time of the upper extremities 0 min, and vitality index 4/10).

During the initial OT assessment (68-69 days after admission), the patient was bedridden all day (Figure 2). Range of motion (ROM) was limited to the shoulder, elbow, and wrist joints. We used the Medical Research Council (MRC) scale, which is a manual muscle strength assessment [14] to evaluate the muscle weakness of the upper extremities. The scale is a useful and quick tool to quantify muscle weakness in ICU-AW [15]. The MRC-sumscore is a summation of the strength of six muscle groups (abduction of the arm, flexion of the forearm, extension of the wrist, flexion of the leg, extension of the knee, and dorsal flexion of the foot) tested on bilateral sides according to the MRC scale (0: no visible contraction; 1: visible contraction without movement of the limb; 2: movement of the limb but not against gravity; 3: movement against gravity over the full range; 4: movement against gravity and resistance; and 5: normal). The MRC-sumscore ranges from 0 (tetraplegia) to 60 (normal strength). The sumscores of <48 and <36 indicate ICU-AW and severe ICU-AW, respectively [16]. Because we tested only three muscle groups of upper extremities at bilateral sides, the MRC score ranges from 0 to 30. The MRC score in our case was 6/30. Grip and pinch strengths (right/left) were 0/0 kgf. ADLs were assessed using the Functional Independence Measure (FIM), where a higher score indicates greater independence [17-19]. The FIM self-care item score was 6/42. The active time of the upper extremities was 0 min, except during the individual rehabilitation program; therefore, he could not manipulate the nurse’s call. Vitality index was 4/10. The primary goal of OT was to improve the FIM self-care item score. The secondary goals were to increase the activity time of the upper extremities and to strengthen the muscles in order to achieve the primary goal.

The intervention was divided into three phases. PSB-based training was conducted five times per week, with each session lasting 20 to 40 minutes. A total of 30 sessions were performed until the patient achieved an MRC score of 3 or higher. The level of PSB assistance was adjusted daily according to the patient’s muscle strength. Neither delirium nor cognitive decline was observed on the Confusion Assessment Method-ICU or Raven’s Colored Progressive Matrices (RCPM) during the intervention period.

Independently pressing the nurse’s call (days 70-87)

**Figure 3:**
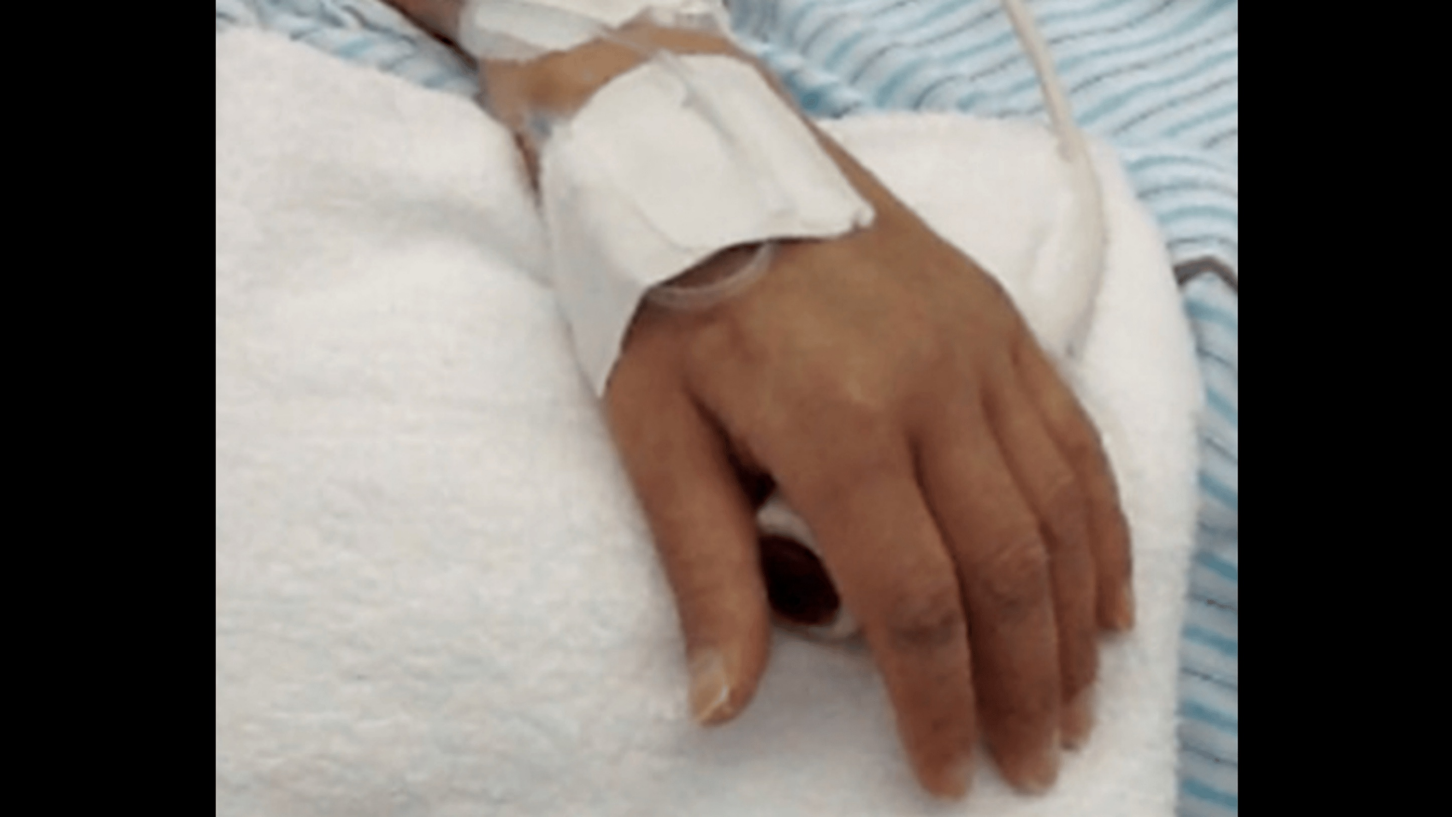
Intervention for manipulating the nurse’s call. Handle of the nurse call was made thicker because the patient could not grasp the usual nurse call (default). A towel was placed as the non-slip instrument (the dotted black arrow) because the patient could not re-grasp the nurse’s call.

Active-assistive ROM exercises for the upper extremities and hands were performed in bed because of a history of VT/VF and CRP level of 17.4 mg/dL. Although active ROM of the hand improved, the patient was unable to grasp or push the nurse’s call owing to muscle weakness. We made the handle of the nurse’s call thicker and he was able to manipulate it (Figure 3).

Feeding and grooming with minimal assistance (days 88-108)

**Figure 4:**
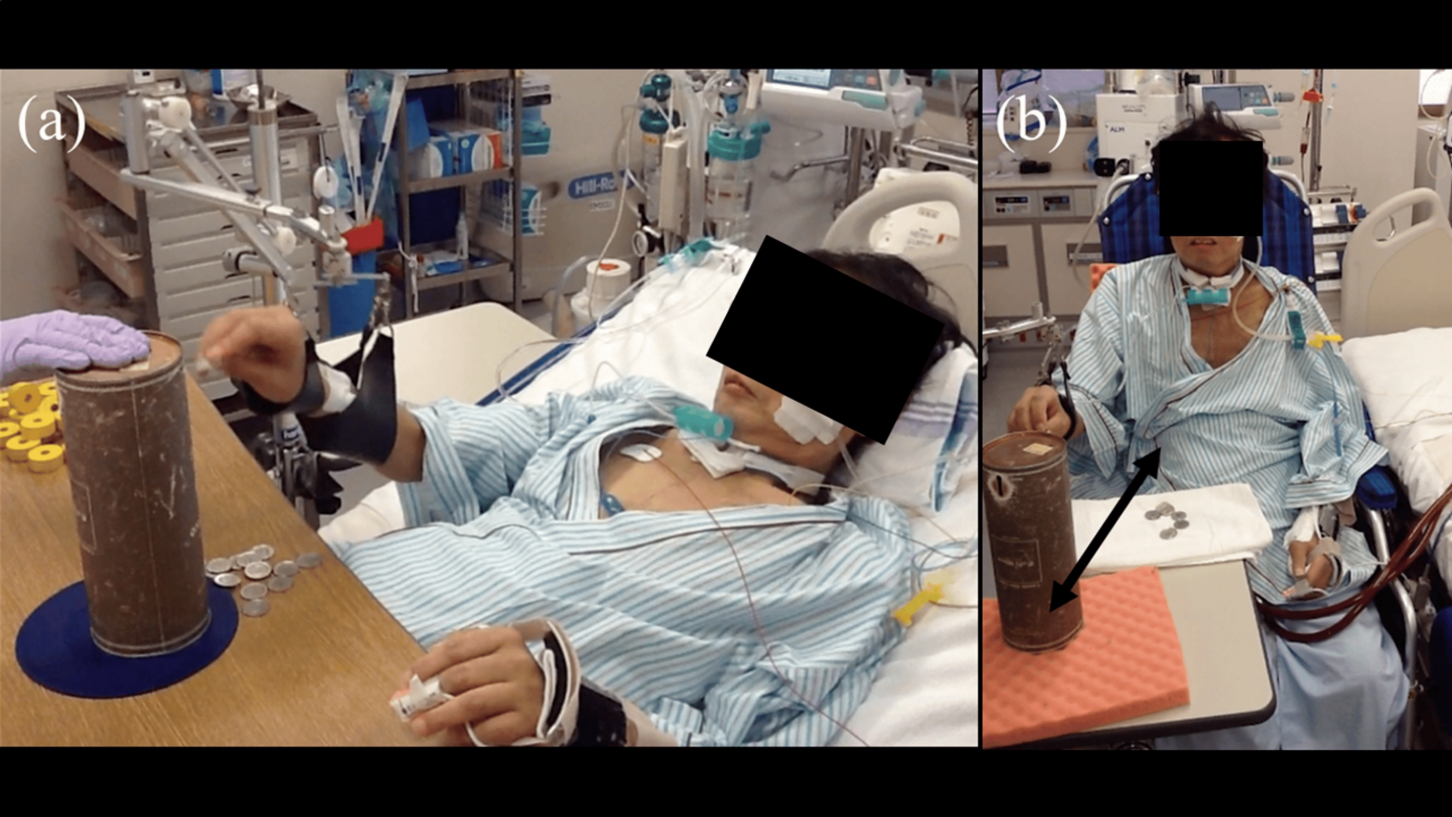
Goal-directed functional training for the upper extremities using the portable spring balancer. In goal-directed functional training, the amount of assistance by the portable spring balancer (the dotted white arrows) was adjusted to refer to fatigue. (a) Training with the head-up 30°. Position increases the stability of the trunk and allows easy movement of the right upper extremities in comparison with sitting on a chair. (b) Training while seated in the wheelchair. Compared to the position of the head-up 30°, training while seated on the wheelchair induces a greater burden on the respiratory circulatory responses because the patient is required to hold his posture statically and dynamically. Distance between the patient and the reaching point (the black two-way arrow) was adjustable to change the trained muscles (i.e., the shorter distance for the distal parts and the longer distance for the proximal parts of the upper extremities).

**Figure 5:**
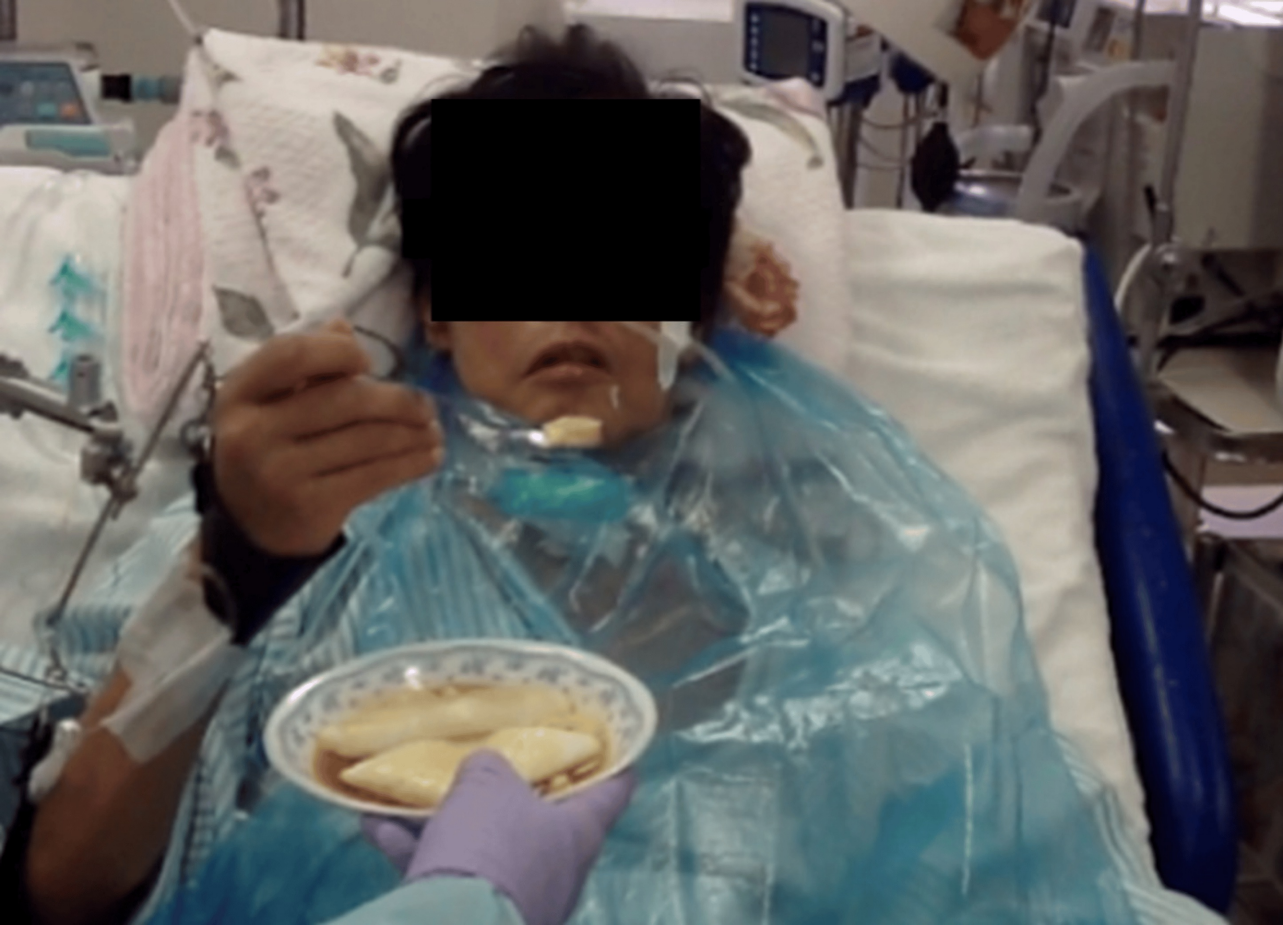
Feeding with minimal assistance. The patient needed assistance part-way through the feeding due to the reduced muscle endurance.

Feeding and grooming were selected as the primary goals because they can be performed while sitting on the bed. To achieve this, functional exercises for the upper extremities were performed horizontally and vertically using a PSB while sitting on a bed or wheelchair (Figure 4). The therapist varied the difficulty of the task (e.g., the angles of the head-up) each day based on medical findings and fatigue. The patient repeatedly touched his lips using the PSB on day 108. Moreover, he could perform feeding and grooming under the condition of the use of the PSB and minimal assistance (Figure 5). The upper extremities MRC score was 15/30 points (shoulder 2/2, elbow 3/2, hand 3/3), motor FIM was 24/91 points, and the self-care FIM was 12/42 points (eating 4, dressing 4, bathing 1, upper dressing 1, lower dressing 1, toileting 1).

Feeding and grooming with modified independence (days 109-125)

**Figure 6:**
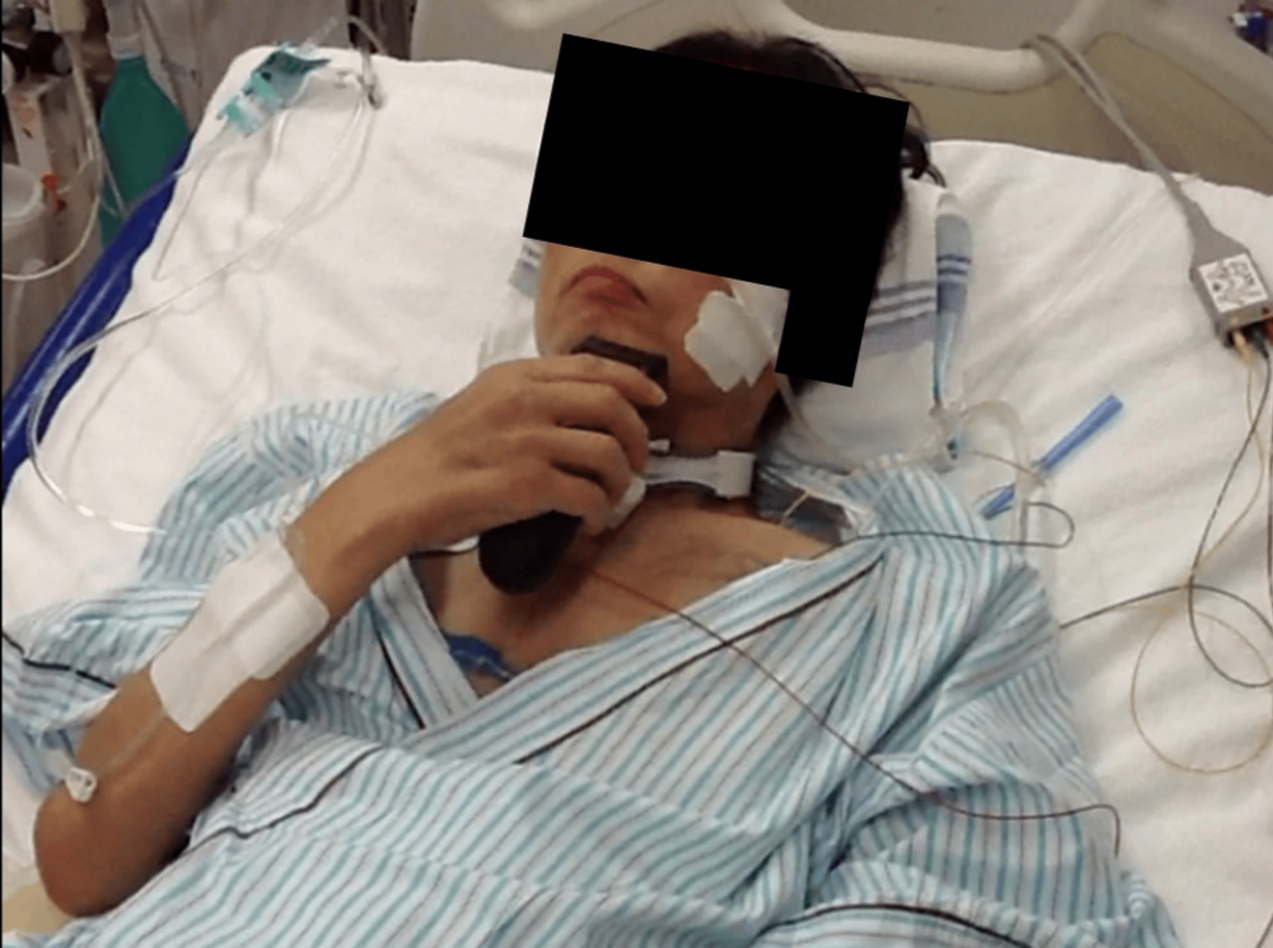
Grooming (shaving) with modified independence. When the electric shaver was put on the bedside table, the patient could shave regardless of the head-up angle.

The patient did not complete feeding or grooming due to fatigue. Therefore, the patient started goal-directed exercises (i.e., mimicking eating and grooming movements) with nurses beyond OT. On day 125, he could feed and groom himself with a PSB (Figure 6). Both feeding and grooming achieved FIM scores of 6 points. The upper extremities MRC score was 20/30 points (shoulder 2/2, elbow 4/4, hand 4/4), the motor FIM was 32/91 points, and the self-care FIM was 20/42 points (feeding 6, grooming 6, bathing 2, upper dressing 2, lower dressing 2, toileting 2).

The final assessment, performed on days 126-127, demonstrated an improvement in the upper extremities MRC score from 6 to 20 out of 30, indicating a 233% increase. Grip, lateral pinch, and pulp pinch strengths (right/left) were 6.7/2.7, 1.8/1.1, and 1.1/0.9 kgf, respectively. The FIM self-care item score improved from 7 to 20 out of 42. This indicates a 185% increase over the course of intervention. Following the intervention using the PSB, the upper extremities MRC score improved from 6 to 20, and the FIM self-care score improved from 6 to 20. This suggests a notable improvement in upper extremities strength and self-care independence. Therefore, activity time of the upper extremities was 50 minutes, except during the individual rehabilitation program. Vitality index was 10 out of 10. All outcomes improved compared with those of the initial assessment (Table 1). A therapist introduced the nurse to handle the PSB.

**Table 1:** Initial and final assessments. *Total score was 30 points because only the bilateral upper extremities were calculated. Score for each body part ranged from 0 (complete paralysis) to 5 (normal strength). R / L, right / left

	Initial assessment	Final assessment
Number of days after admission	70–71	126–127
Muscle strength		
MRC score, point^* ^(shoulder, elbow, hand, R / L)	6 (1 / 1, 1 / 1, 1 / 1)	20 (2 / 2, 4 / 4, 4 / 4)
Grip strength, kgf, R / L	0.0 / 0.0	6.7 / 2.7
Lateral pinch, kgf, R / L	0.0 / 0.0	1.8 / 1.1
Pulp pinch, kgf, R / L	0.0 / 0.0	1.1 / 0.9
Functional Independent Measure		
Self-care items, point	6	20
Feeding	1	6
Grooming	1	6
Bathing	1	2
Dressing (upper body)	1	2
Dressing (lower body)	1	2
Toileting	1	2
Others		
Activity time of the upper extremities, minute/day	0	50
Vitality index, point	4	10

## Discussion

This patient had severe ICU-AW but showed improvements in muscle strength and ADL through repeated, goal-directed OT assisted by a PSB, along with appropriate environmental modifications. Although there have been reports of several interventional studies targeting critically ill patients, this case differs in terms of patient characteristics and severity. Schaller et al. [20] conducted a multicenter, international, parallel-group, assessor-blinded randomized controlled trial that compared early goal-directed mobilization, which incorporated a multidisciplinary approach and utilized the SICU Optimal Mobilization Score (SOMS) algorithm, with standard care. The study enrolled patients aged ≥18 years who had been mechanically ventilated for ≤48 hours and were expected to require continued ventilation for at least an additional 24 hours. Compared to their cohort, the patient in our case was younger (52 years vs a median age of 66 years) and had greater disease severity (APACHE-2 score of 28 vs a median of 16).

PT was initiated on day 19. However, OT and ST were delayed until days 68 and 70, respectively, due to the patient's hemodynamic instability and use of sedatives. Although PT was provided prior to OT, it mainly focused on early mobilization and general mobilization rather than upper extremity function. Therefore, the improvements observed in upper limb strength and ADL performance during the PSB intervention period were likely due to the OT intervention. During the OT period, no delirium or cognitive impairment was observed based on the CAM-ICU and RCPM, suggesting minimal influence of sedation. These observations suggest that the potential confounding effects of concurrent rehabilitation therapies and fluctuating sedation levels were minimal. PSB was developed to help patients with muscle weakness perform ADL, such as feeding and writing [21]. The PSB holder was mounted on a three-dimensional arm with a spring-loaded post. Patients can perform reaching movements because the upper extremities supported by spring tension through the cuff on the elbow and wrist and the effect of gravity decrease. This case demonstrates that even patients with severe ICU-AW can actively engage in upper extremity movements and participate in repeated, goal-directed training using a PSB.

A meta-analysis showed no significant differences in isometric muscle strength between low- and high-load resistance training [22]. The PSB allows the load to be adjusted by adjusting the springs. Therefore, repeated goal-directed upper extremities functional training may have facilitated muscle strengthening in this case.

Intervention in the environments by OT and collaboration with nurses could increase the activity time of the upper extremities in the ICU. Consequently, further increases in muscle strength and endurance may occur.

ADL in patients admitted to the ICU can be improved by early rehabilitation [23] and goal-directed rehabilitation [20]. Our findings are consistent with these findings and suggest a novel strategy for ICU rehabilitation.

While the improvements in muscle strength and ADL observed in this case are encouraging and consistent with the before-and-after data, it is important to acknowledge that this report describes a single case without a control group. Therefore, causal inferences should be made with caution. The potential for selection bias also limits generalizability. To validate the effectiveness of PSB-based, goal-directed OT for ICU-AW, future studies with more rigorous designs are warranted, including pilot randomized controlled trials that compare this intervention to standard care.

## Conclusions

This case report provides the strategy and results of an OT intervention for severe ICU-AW. Exercise for physical functions and intervention in the environment, such as the use of welfare equipment and human resources, potentially contributes to improving muscle weakness and limiting ADL. This approach is considered applicable in various ICU settings due to the ease of PSB installation. This case report provides a basis for a study examining the effectiveness of rehabilitation in ICU-AW patients. As a future perspective, a multicenter randomized controlled trial is planned to facilitate generalization.
